# Protocols for Analyzing the Role of Paneth Cells in Regenerating the Murine Intestine using Conditional *Cre-lox* Mouse Models

**DOI:** 10.3791/53429

**Published:** 2015-11-21

**Authors:** Lee Parry, Madeleine Young, Fatima El Marjou, Alan Richard Clarke

**Affiliations:** ^1^European Cancer Stem Cell Research Institute, Cardiff University; ^2^Institut Curie

**Keywords:** Developmental Biology, Issue 105, Intestine, Cre-Lox, mouse, stem cell, Paneth cell, transgenic

## Abstract

The epithelial surface of the mammalian intestine is a dynamic tissue that renews every 3 - 7 days. Understanding this renewal process identified a population of rapidly cycling intestinal stem cells (ISCs) characterized by their expression of the *Lgr5 *gene. These are supported by a quiescent stem cell population, marked by *Bmi-1 *expression, capable of replacing them in the event of injury. Investigating the interactions between these populations is crucial to understanding their roles in disease and cancer. The ISCs exist within crypts on the intestinal surface, these niches support the ISC in replenishing the epithelia. The interaction between active and quiescent ISCs likely involves other differentiated cells within the niche, as it has previously been demonstrated that the ‘‘stemness’’ of the *Lgr5* ISC is closely tied to the presence of their neighboring Paneth cells. Using conditional *cre-lox *mouse models we tested the effect of deleting the majority of active ISCs in the presence or absence of the Paneth cells. Here we describe the techniques and analysis undertaken to characterize the intestine and demonstrate that the Paneth cells play a crucial role within the ISC niche in aiding recovery following substantial insult.

**Figure:**
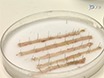


## Introduction

The luminal surface of the mammalian intestine features repeating units of crypts and finger like projections, termed villi, which protrude into the lumen. This surface is a continuous sheet of epithelia which undergoes complete self-renewal approximately every 3 - 4 days^1^. This dynamic tissue is supported by a population of rapidly cycling stem cells (ISCs; also known as crypt base columnar cells), which were initially identified by their expression of the *Lgr5 *gene^2,3^. These cells exist in a specialized niche at the bottom of the crypts of Lieberkuhn. Initially, the discovery that ISCs were rapidly cycling was discordant with the prevailing idea that a stem cell was quiescent in nature. Previous to the identification of the *Lgr5^+ ^*ISC it was postulated that a population of quiescent label retaining cells at the +4 position, relative to the base of the crypt, were the ISCs^1^. Recent research has now reconciled these observations by demonstrating that primarily there is a pool of equipotent cycling ISCs in each crypt whose fate are regulated by its neighbors^4,5^. In the event they are lost these can be replaced by quiescent cells that ordinarily are committed to the secretory lineage but can revert to ISCs if the ISC population is damaged^6^.

ISC neighbors can either be ISCs or their daughter cells. The ISCs produce naïve daughter cells which multiply and differentiate into the specialized cell types that comprise the epithelial sheet which lines the intestinal lumen^1^. The goblet, enteroendocrine, enterocytes, tuft and M cells migrate upwards to the luminal surface where they provide various absorptive and regulatory functions, however, the Paneth cells remain at the bottom of the crypt where they exist intermingled with the ISCs. In recent years it has been demonstrated that a proportion of the naïve daughter cells destined for a secretory lineage are quiescent label retaining *Lgr5*^lo^ cells capable of reverting to an ISC upon injury^6,7^.

Due to its importance in crypt regeneration a priority was placed on understanding the interactions between the ISCs and its neighbors, particularly the Paneth cells. The Paneth cells play a crucial role in the niche which supports the ISCs^8^. In addition to bactericidal products the Paneth cells produce signaling molecules that activate the pathways which govern ISC renewal or differentiation. Previous studies showed that the *Lgr5*^+^ ISCs could only exist when they could compete for essential niche signals provided by their daughter Paneth cells^8^. These studies examined the role of Paneth cells on normal *Lgr5*^+^ ISCs and not in a situation where they are damaged and require replenishment from an *Lgr5^lo ^*population.

To understand intestinal biology and model disease we examine the functional role of cells and/or genes using transgenic mouse models^9,10^. Frequently these models utilize *cre-lox* technology to conditionally modify gene(s)^9,10^. *Cre* (Causes recombination) recombinase is a site specific recombinase of the integrase family, isolated from bacteriophage P1. *Cre* catalyses site specific recombination between defined 34 bp ‘Lox P’ (locus χ of crossover P1) sites. Mice are genetically engineered to contain *LoxP *sites that flank regions of interest which upon expression of *Cre *recombinase are excised. Linking the expression of the *Cre *gene to a cell or developmental specific promoter allows for alteration to be made in a spatial fashion^9,10^, this is especially useful in overcoming embryonic lethal mutations. Further linking the *Cre* expression to a receptor pathway, that can be activated artificially, permits temporal alterations.

Using this technology we inactivated the *CatnB *gene^11^ in the intestinal epithelia. β-catenin, the *CatnB *gene product, is a key regulator of the canonical Wnt signaling pathway which governs ISC homeostasis. Two previous studies using this strategy produced conflicting results^12,13^. The study by Fevr *et al.^12^, *demonstrated loss of stem cells and intestinal homeostasis. Whereas the Ireland *et al.^14^*study reported that following a reduction in cell viability the crypt-villus axis was repopulated from wild type cells expressing *CatnB*. The major difference in these studies was the promoter used to express *Cre* in the intestinal epithelia. The Fevr *et al.*, study used the villin gene promoter linked to the estrogen receptor which can be activated by administering tamoxifen (*vil-Cre-ER^T2^*)^15,16^. In contrast Ireland *et al.*, utilized the promoter element of the rat cytochrome P450A1 (*CYP1A1*) gene to drive *Cre* expression in response to the xenobiotic β-naphthoflavone (*Ah-cre*). The characteristics of these different systems generated two hypotheses to account for these different observations. The first that *CatnB *is more efficiently deleted in the ISC using the *vil-Cre-ER^T2 ^*system compared to the *Ah-cre*, thereby reducing the number of ISCs to sub-repopulation levels. Alternatively it was due to differential *CatnB *deletion in the differentiated cell population. The *vil-Cre-ER^T2 ^*system targets all epithelial cells of the crypt and villus whereas the *Ah-cre *system only targets the non-Paneth cells of the ISC niche and crypt. These systems provided ideal tools for examining the behavior of the ISCs and their interaction with the Paneth cells. Here we present several detailed protocols based on how we used these systems to determine that Paneth cells play a crucial role in mediating the intestinal response to injury^17^.

## Protocol

Information on all material used is given in **Table 1**. All animal experiments were performed under the authority of a U.K. Home Office project license.

### 1. *CatnB *Deletion using the *Ah-cre* and *vil-Cre-ER^T2^* Systems

1. Cross the mice strains to generate 10 - 14 week old cohorts of *Ah-cre^+^*CatnB^+/+^, *Ah-cre^+^*CatnB^flox/flox^, *vil-Cre-ER^T2^CatnB^+/+^* and *vil-Cre-ER^T2^CatnB^flox/flox^*.  For visual analysis of recombination, via a β*-gal­* stain, cohorts should contain the *Rosa26R-lacZ * reporter^17^.  Cohorts should control for the presence of modified genes and use of induction agents, the size of cohorts required should be estimated using a power analysis.
2. To induce the *Ah-cre *transgene prepare β-Naphthoflavone (BNF), or for the *vil-Cre-ER^T2 ^*transgene tamoxifen (TAM) in corn oil to give a working solution of 10 mg/ml. NOTE: The agents should be weighed out in a fume hood using appropriate personal protection.
3. Heat solutions in an amber bottle (BNF is light sensitive) to either 99.9 °C for BNF or 80 °C for TAM in a water bath.
4. Transfer to a heated stirrer set at 100 °C for BNF, or 80 °C for TAM, and stir for 10 min.
5. For BNF repeat 1.3-1.4 until dissolved (can take >1 hr).
6. Aliquot into small amber bottles (~5 ml) and then freeze at -20 °C.  Bottles should be discarded after 3 freeze/thaw cycles.  Prior to use thaw agents and reheat to appropriate temperature if they have fallen out of solution.  Allow to cool to <37 °C prior to injection.
7. Inject the mice intraperitoneally (I.P.) with a dose of 80 mg/kg, for example a 25 g mouse receives 0.2 ml of the appropriate induction agent. For *Ah-cre* deliver three injections in 24 hr period, for *vil-Cre-ER^T2^* give one injection per day for 4 days.

### 2. Dissection of Intestine for Reporter Visualization and Immunohistochemistry (IHC)

1. Euthanize the mouse using cervical dislocation without prior anesthesia in accordance with ethical approval. Place mouse in a supine position and wet the fur using 70% EtOH, open the intra-peritoneal cavity longitudinally along the midline using a scissors.
2. Secure the stomach with a forceps and sever the connection to the esophagus. Remove the small intestine up to the appendix by gently pulling on the stomach. Remove the large intestine up to the anus by gently pulling on the appendix.
3. Once the intestines have been isolated remove the stomach and appendix. Flush intestines with 1x PBS using a syringe with a blunt ended pipette tip.  NOTE: Each intestine should be** immediately** processed for one of the downstream applications described below.

### 3. Formalin Fixation of Intestine

1. Cut the flushed intestine into 3 equal sized sections and label proximal, middle and distal. Cut each section into 1 cm pieces. Take a small strip of surgical tape 2 cm x 2 cm. Place three to five 1 cm pieces on to the middle of the surgical tape in a pyramid formation.
2. Close and seal the tape around the pieces longitudinally, to give a “log pile” effect. Place tissue in a flat bottomed container containing a large excess of neutral buffered formalin fixative, at least 10x the volume of fixative to the volume of tissue.  NOTE: Avoid placing excess amounts of tissue within a tube for fixation, divide it into multiple containers.
3. Place samples at 4 °C for at least 18 - 24 hr prior to embedding and sectioning.  To prevent loss of nuclear β-catenin, do not fix beyond 24 hr. After fixation transfer tissue to a flat bottomed container containing a large excess of 70% EtOH, at least 10x the volume of tissue.

### 4. Methacarn Fixation of Intestine

1. Prior to dissection prepare methacarn fixative by combining 300 ml MeOH, 150 ml chloroform and 75 ml glacial acetic acid (4:2:1). Cut the flushed intestine into 3 equal sized sections proximal, middle and distal.
2. Place each piece of intestine side by side on a piece of filter paper (15 cm x15 cm) and using a springbow scissors open it up ‘en face’. Place the intestine and filter paper into a glass dish containing methacarn for 3 - 24 hr at RT. After fixation pick up the end of an intestine section using a forceps.
3. Wind the intestine around the forceps to form a “swiss roll” and secure the roll by slightly opening the forceps and putting a 25 G needle through it.   Place tissue in a flat bottomed container containing a large excess of neutral buffered formalin fixative, at least 10x the volume of fixative to the volume of tissue and store for at least 1 hr before proceeding to processing.

### 5. Whole Mount LacZ Visualization (Modified from El Marjou *et al.^18^*)

1. Prepare wax plates by combining molten ralwax with mineral oil at 10:1. Pour into 15 cm petri dishes and leave to cool. Prepare X-gal fixative as per **Table 1** and store on ice.
2. Remove whole intestine and flush through with ice-cold 1x PBS, as per section 2. Fix intestine by flushing with 25 ml ice-cold X-gal fixative.
3. Using a scissor cut the intestine into 3 - 5 equal sections (maximum 5 per plate).  Place each section onto wax plate and pin down each end so the section is slightly stretched with the mesenteric line uppermost; trim any excess mesentery. Using a springbow scissors cut the gut longitudinally and pin out along the way. 
4. Flood the plate with X-gal fixative to cover the sections and leave for at least 1 hr at 4 °C. Remove X-gal fixative using a 25 ml pipette and wash once with 30 ml of 1x PBS. Cover sections with 30 ml of DTT demucifying solution for 30 - 60 min at RT, ideally on a rocking platform.
5. Remove demucifiying solution using a 25 ml pipette and flood the plate with 30 ml of 1x PBS.  Using a pasteur pipette wash the intestine sections with the 1x PBS in the plate to remove mucus.
6. Remove 1x PBS with a 25 ml pipette and flood with 30 ml of X-gal stain. Incubate overnight at RT in the dark with gentle agitation on a rocking platform.
7. Following the overnight incubation check that the sections have developed a blue/green stain, if the background color is still white, fresh staining solution can be added and monitored till staining develops. NOTE: Once sections have stained then no further staining can be attempted.
8. Remove the X-gal stain using a 25 ml pipette and flood plate with 30 ml of 1x PBS and leave for 3 min with gentle agitation.  Remove pins and pick up, with forceps, the end of an intestine section. Wind the intestine around the forceps to form a “Swiss roll”, secure the roll by slightly opening the forceps and putting a 25 G needle through it.
9. Place tissue in a wide mouthed flat bottomed container containing a large excess of neutral buffered formalin fixative, at least 10x the volume of fixative to the volume of tissue.   Place samples at 4 °C for at least 24 hr prior to embedding and sectioning. 

### 6. Extraction of Crypts from Intestine

1. Isolate the first 20 cm of the small intestine, as per section 2. Place the intestine on a clean dissecting surface and using a forceps and scissors remove any attached fat/mesentery. Using a springbow scissors open the gut longitudinally.
2. Using a microscope cover slide, firmly scrape the gut lumen to remove villi and mucus. Using a scissors cut the intestine into ~5 mm pieces and transfer into a 50 ml tube with 25 ml 1x HBSS supplemented with penicillin (100 U/ml) and streptomycin (100 U/ml). 
3. Incubate for 10 min at RT. Remove the antibiotic containing media by passing the samples through a 70 µm cell strainer.
4. Place intestinal sections into a fresh 50 ml tube containing 10 ml 1x HBSS and replace the cap.
5. Gently invert twice and remove the 1x HBSS by passing through a 70 µm cell strainer. Further wash the intestinal pieces by repeating 6.4 & 6.5  three times and ensure that the final fraction is relatively clear.
6. Transfer tissue to a fresh 50 ml tube containing 10 ml of EDTA (8 mM)/1x HBSS and leave at RT for 5 min. Shake vigorously (20 - 30x) or vortex, pass through a 70 µm cell strainer and transfer tissue pieces to a fresh 50 ml tube containing EDTA (8 mM)/1x HBSS.  NOTE: The flow through can either be discarded or retained if analysis of villi epithelia is required.
7.  Incubate the tissue pieces on ice for 30 min, shake the sample vigorously (20 - 30x) or vortex. Pass the samples through a 70µm cell strainer and retain the flow through as this contains the crypts.
8.  Transfer the tissue pieces to a fresh 50 ml tube containing 10 ml of 1x HBSS.  Shake vigorously (20 - 30x) or vortex, pass through a 70 µm cell strainer and retain the flow through. Repeat one more time to ensure maximum recovery of crypts from intestinal pieces. Combine the flow through fractions and centrifuge at 300 x g for 5 min. Pour off the supernatant and retain the crypt pellet.  NOTE: The pellets can be used immediately for culturing (if applicable) or stored at -80 °C prior to standard DNA/ RNA/protein extraction procedures.

### 7. Standard Immunohistochemical Visualization

1. Cut 5 µm sections of paraffin embedded tissue onto poly-L-lysine (PLL) slides. Note: A standard protocol for staining with a B-catenin antibody is given below, parameters for other antibodies are given in **Table 2**.
2. De-wax with 2x 3 min washes in slide baths containing fresh xylene. Rehydrate by passing slides for 3 min through slide baths containing fresh: 100% EtOH (2x), 95% EtOH and 70% EtOH and finally into 1x PBS.
3. Place slides into a slide bath containing citrate buffer (pH 6) and heat 99.9°C for 20 min to retrieve antigens. Allow slides to cool and then wash 3x 5 min in a slide bath containing 1xTBS/T for 5 min. Remove slides from last wash, immediately draw around tissue with a PAP pen, and cover section with a commercial peroxidase block or 1.5% H_2_O_2 _(in distilled H_2_O).
4. Incubate for 20 min at room temperature (RT) then wash 3x in slide baths containing fresh 1x TBS/T for 5 min. After washing cover sections, using a pipette, in 5% Normal Rabbit Serum (NRS)/1xTBS/T for 30 min at RT to block non-specific hydrophobic binding of your primary antibody.
  1. Remove NRS block with a Pasteur pipette and cover section in B-catenin primary antibody diluted 1:200 with 5% NRS. Wash slides for 3x 5 min in slide baths containing fresh 1xTBS/T. Note - other antibodies will require optimization for dilution and specificity.  To ensure specificity of an antibody, appropriate no antibody and isotype control stains should be performed.  An isotype control is matched to the host species and isotype of your primary antibody.
5. Visualize with either a commercial HRP detection kit or with an appropriate fluorescently labelled secondary antibody (**Table 2**).  NOTE: Length of development differs for each antibody, for β-catenin 10 - 15 sec is usually sufficient.  To optimize development for an antibody first establish the length of time required to visualize positive cells using a positive control slide and then apply this to all subsequent slides.
6. Wash slides for 3x 5 min in slide baths containing fresh TBS/T. Counterstain slides by immersing in a slide bath containing haematoxylin for ~45 sec (not required if using fluorescently labelled secondary antibodies).  Put slides into a clean slide bath and rinse with running tap water for ~1 min, ensuring the haematoxylin is not completely washed out.
7. Dehydrate slides by passing through slide baths containing increasing concentrations of alcohols; 1x 30 sec  in 70% EtOh, 1x 30 sec in 95% EtOH, 2x 30 sec washes in 100% EtOH, 2x 2 min in xylene.
8. Mount slides under a coverslip using a commercial mounting media. NOTE: If using fluorescently labeled antibody mount with a media containing DAPI to label the nucleus.

### 8. Histological Identification of Specific Intestinal Epithelial Cells

1. Enterocytes
  1. Prepare sections using section 7 with the villin antibody and conditions described in **Table 2**.
2. Entero-endocrine cells (Grimelius stain ^18,19^).
  1. Prepare sections by following steps 7.1-7.2. Wash slides in a slide bath containing ultrapure water for 3 min. Transfer the slides to a slide bath containing preheated silver solution (**Table 1**) and incubate at 60 °C for 3 hr.
  2. Remove the slides from the silver solution and place in a slide bath containing freshly prepared preheated reducer solution (**Table 2**) at 45 °C for 3 min.  Remove the slides and place into a slide bath containing fresh ultrapure water for 3 min. Follow steps 7.6-7.8 from section 7.
3. Goblet cells (Alcian Blue stain).
  1. Prepare sections by following steps 7.1-7.2.. Transfer slides to a slide bath containing Alcian Blue pH 2.5 for 5 min at RT.  Remove Alcian blue stain with a pipette and place slide bath under a running tap for 3 - 5 min. Follow steps 7.6-7.8.  NOTE: Alcian blue solution can be retained for further use.
4. ISCs.
  1. To identify the ISCs follow section 9.
5. Paneth cells.
  1. Prepare sections following section 7 using the lysozyme antibody and conditions described in **Table 2**.

### 9. *In Situ* RNA Detection with the Murine Intestine^19-21^

1. Place 5 µm sections from formalin fixed intestine (section 3) onto PLL slides.   Prepare a linearized digoxigenin labelled RNA probe for detecting *Olfm4 *expression*^21^*. Dewax and rehydrate sections as per section 7. NOTE: This protocol should be performed in an RNAse free environment to prevent degradation of the RNA probe.
2. Draw around tissue with a PAP pen to minimize reagents. Incubate sections in a slide bath with 6% H_2_O­_2 _(in distilled H_2_O) for 30 min.  Wash twice in a slide bath with fresh 1x PBS for 3 min. Discard 1x PBS and, using a pipette, cover section with 4% paraformaldehyde for 20 min on ice.
3. Wash twice in a slide bath with fresh 1x PBS for 3 min. Using a pipette cover sections with Proteinase K solution for 5 min  Wash in a slide bath with fresh 1x PBS for 3 min. Using a pipette post-fix sections by covering in 4% paraformaldehyde for 5 min at RT.
4. Wash in a slide bath with DEPC treated H_2_0 for 2 min. Using a pipette cover sections in acetic anhydride solution for 10 min with agitation. Wash in a slide bath with fresh 1x PBS/3 min, followed by 1x saline/3 min. Pass slides through baths containing increasing concentrations of alcohols; 1x 30 sec  in 70% EtOh, 1x 30 sec in 95% EtOH, 2x 30 sec washes in fresh 100% EtOH, 2x 2 min in fresh xylene and allow to air dry.
5. Dilute *Olfm4* probe 1:100 in hybridization buffer and denature probe by heating to 80˚C for 3 min. Apply 100 μl of probe to each section and cover with parafilm to prevent dehydration of the slide.  Incubate overnight in a dark, moist chamber at 65 ˚C.
6. Wash in a slide bath with 5× SSC at 65 ˚C for 15 min. Wash sections in a slide bath twice with fresh 50% formamide/5× SSC/1% SDS for 30 min at 65 ˚C. Wash sections twice in a slide bath in fresh PBT for 10 min, the first at 65 ˚C and the second at RT. Using a pipette cover sections with PBT containing 25 µg RNAse for 45 min at 37 ˚C. Wash sections in a slide bath in PBT for 5 min at RT.
7. Wash sections in a slide bath twice with fresh 50% formamide/5× SSC for 30 min at 65 ˚C. To block, cover sections using a pipette with 10% sheep serum in PBT and store in a dark, moist chamber at RT for 2 - 3 hr.
8. Prepare antibody by diluting anti-digoxigenin alkaline phosphatase conjugated antibody at 1:500 with 10% sheep serum in PBT containing 5 mg/ml mouse intestinal powder. Incubate for 3 hr at 4 °C in dark on a rocking platform. Spin down to remove excess intestinal powder and add 3x volumes of 1% sheep serum in PBT to the supernatant.
9. Remove block from slides with a pipette and add 100 μl of the antibody solution to each section, cover with parafilm and incubate in a dark, moist chamber at 4°C overnight. Wash sections in a slide bath 3× with fresh PBT for 5 min. To block sections wash in a slide bath 3× with fresh NTMT buffer for 5 min.
10. To visualize, using a pipette cover each section with BM purple and incubate in the dark at RT for 24 - 72 hr until a sufficiently strong colour develops. Wash sections in a slide bath once in PBT and counterstain by immersing in eosin for 1 min.  Remove excess eosin by washing sections in a slide bath under a running water for 3 - 5 min. Immerse slides in xylene and allow to air dry. Mount under a coverslip using commercial media.

### 10. Histological Characterization of Intestinal Epithelia

1. Place 5 µm sections from the fixed tissue (section 3 & 4) onto PLL slides.  Analyze ≥25 random whole (or ≥50 half) crypts from ≥4 mice of each cohort for each of the following parameters.  NOTE: To maintain consistency analyze crypts from the same location of each intestine (only use the proximal end).
2. Cellular parameters from standard H&E stained sections (unless otherwise stated): Crypt number, height, apoptosis and mitosis.
  1. Crypt number.
    1. Use a low power magnification (*e.g.* 4X or 10X) to manually count the number of crypts in contact with the basal layer.  Count ≥10 transverse proximal intestine sections from at least 4 mice.
  2. Crypt height.
    1. Use a high power magnification (*e.g.* 20X or 40X) to manually count the number of cells from the bottom of the crypt to the crypt/villus axis (**Figure 3b**).
  3. Apoptosis^22^^,^^23^.
    1. Method 1: Use a high power magnification (*e.g*. 20X or 40X) to manually count the numbers of apoptotic cells in each crypt.  Apoptotic cells can be identified by cell shrinkage, chromatin condensation, formation of cytoplasmic blebs and apoptotic bodies (**Figure 3a**).
    2. Method 2: Perform an IHC stain (section 7) for Caspase-3 using conditions described in **Table 2**.  Using a high power magnification (*e.g*. 20X or 40X) manually count the number of positive cells in each crypt to quantitate the cells in the execution phase of apoptosis.
  4. Mitosis.
    1. Use a high power magnification (*e.g.* 20X or 40X) to  manually count the numbers of mitotic cells in each crypt.  Mitotic cells contain condensed DNA material and are typically symmetric and well formed (**Figure 3b**).
  5. Proliferation.
    1. Perform an IHC (section 7) stain for Ki-67 using conditions described in **Table 2**.  Manually count positive cells to quantitate the proportion of crypt cells proliferating. 

## Representative Results

***Comparing ISC Recombination Efficiency in the Ah-cre and Vil-Cre-ER*** ***^T2^*** ***Systems***

Use of these *cre-lox* systems for evaluating the role of Paneth cells, in repopulating the intestine following damage, required characterization of the efficiency of recombination within the ISCs. Using the *Rosa26R-lacZ *conditional reporter we demonstrated that in both systems 3 days post induction (d.p.i.) there is ~100% recombination in the small intestine (**Figure 1a**). Quantitating the presence of the recombined allele by qPCR was confounded by the differences in *Cre *expression patterns between the systems. The *vil-Cre-ER^T2 ^*system showed a 3.53 fold increase in the presence of the recombined allele compared to the *Ah-cre* system, due to its expression in a greater proportion of the epithelia^16^. To overcome this we adopted a different strategy that allowed us to directly compare the systems. We induced the mice with different induction regimes and analyzed at 30 d.p.i., at which point *LacZ *positive crypts and villus represent an ISC recombination event. Using this approach we demonstrated that in both systems, 3 injections of inducing agent (delivered I.P. at 80 mg/kg in 24 hr), recombined in an equivalent number of ISCs despite initial recombination levels being far greater in the *vil-Cre-ER^T2 ^*system^16^ (**Figure 1b-d**). Further, using DNA extracted from the recombined crypts, qPCR for the recombined alleles demonstrated a non-significant increase in recombination using the *vil-Cre-*ER^T2 ^system, potentially due to the recombination in the Paneth cells not observed using the *Ah-cre *system (**Figure 1d**). Further, staining for epithelia cell types did not indicate any alteration to differentiation pattern, representative images of each cell type investigated is shown in **Figure 2e-2h**.

**Characterization of Intestinal Epithelia following** ***CatnB*** **Deletion**

***Quantification of Crypt Loss***

Characterizing the kinetics of recombination in these *Cre *systems enabled us to analyse the mouse intestine when equivalent numbers of ISCs are recombined. Using the *LacZ *reporter both systems showed complete loss of recombined (blue) cells at 3 d.p.i. (**Figure 2a**). As previously reported three days after deletion of *CatnB* the *Ah-cre *mice showed partial crypt loss, whereas the *vil-Cre-ER^T2 ^*mice demonstrated complete destruction of the crypt/villus axis^13,16,24^ (**Figure 2b-d**).

***Dynamics of Epithelial Repopulation***

Using the techniques above we characterized multiple parameters to enable us to understand this observation. Representative images of the parameters and cell types analyzed using protocols 7 - 10 are given in (**Figure 3a & 3b**). Briefly, the loss of crypts was consistent with the elevated levels of apoptosis displayed in both systems (**Figure 3e**). However the mitosis, proliferation, crypt cellular height, crypt and expression (not shown) data indicated the *Ah-cre *system could recover, presumably due to repopulation by un-recombined ISCs (**Figure 3c**). In stark comparison, the *vil-Cre-ER^T2 ^*failed to recover despite retaining epithelial crypt cells (**Figure 3d**).

***Characterization of Cellular Phenotypes within Crypt***

To understand why the crypts from *Ah-cre *mice could repopulate whereas the *vil-Cre-ER^T2^* couldn’t we characterized the epithelial cells three days after deletion of *CatnB*. Using *in situ *hybridization (section 9) and IHC analysis (section 7, 8 & 10) we demonstrated that the crypt cells in the *vil-Cre-ER^T2^ CatnB^flox/flox ^*mice were non-proliferative and lacked expression of the ISC marker Olfm4, unlike the crypts in the *Ah-cre *mice (**Figure 4c & f**). As the initial characterization had demonstrated that recombination in crypts was equivalent we proceeded to examine the role of the Paneth cells. We performed a dual fluorescent IHC against *CatnB *and *Lyz1* to identify which cells had lost β-catenin and whether they were Paneth cells (**Figure 5a-5c**). As previously described we demonstrated that all crypt cells are targeted using the *vil-Cre-ER^T2 ^*system. In comparison the *Ah-cre *system spared the Paneth cells and the villus epithelia. Further Paneth cells were only observed undergoing apoptosis after *CatnB *deletion using the *vil-Cre-ER^T2 ^*system (**Figure 5d & 5e**).


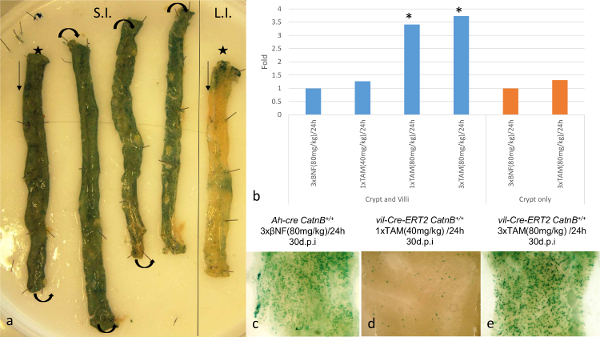
**Figure 1: Comparison of the Specificity and Efficiency of Cre/Lox Recombination within the Intestinal Epithelia using the *****Ah-cre *****and *****Vil-Cre-ER^T2 ^*****Systems.** (a): Visualization of *Ah-creLacZ* reporter expression in wholemount small (S.I.; *distal end) and large (L.I.; *distal end) from a wild type mouse. (b): Results of qPCR showing fold change for the recombined *CatnB^flox ^*allele at 1 d.p.i. to compare different induction regimes in *Ah-cre CatnB^flox/flox ^*(BNF induced) and *vil-Cre-ER^T2 ^CatnB^flox/flox ^*(TAM induced); *P >0.05 (Mann-Whitney [2-tail] compared to control). (c)-(e): Wholemount small intestine showing LacZ positive crypt 30 d.p.i. Panel (b)-(e) modified from Parry *et al.*^16^. Please click here to view a larger version of this figure.


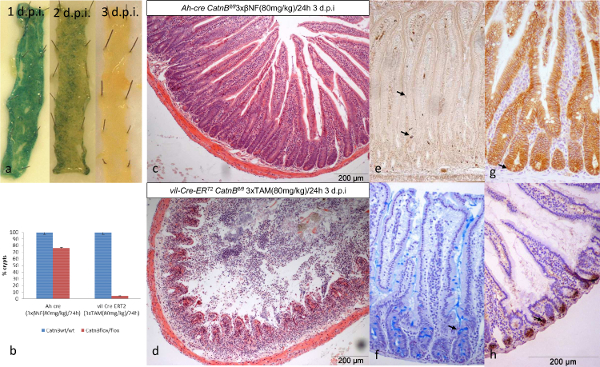
**Figure 2: Comparison of the *****Ah-cre *****and *****Vil-Cre-ER^T2 ^*****Systems for Conditionally Deleting *****CatnB *****in Small Intestine Epithelia.** (a): Wholemount small intestine showing loss of recombined cells in *Ah-cre CatnB^flox/flox^LacZ^+ ^*mice over 3 days. (b): Quantification of crypt loss 3 days after deletion of *CatnB*; *P >0.05 (Mann-Whitney [2-tail] compared to control). (c&d): Transverse H&E sections of formalin fixed intestine demonstrating loss of crypts after *CatnB *deletion. (e)-(h) Example of cell types from control mice: (e) entero-endocriine cells, (f) goblet cells, (g) *CatnB *IHC indicating an ISC (→) with nuclear B-catenin & (h) Paneth cells. Panel (b) modified from Parry *et al.*^16^. Please click here to view a larger version of this figure.


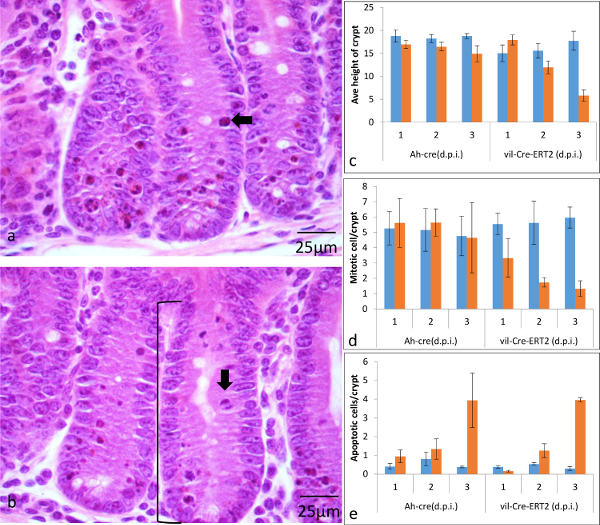
**Figure 3: Characterization of the Onset of Phenotype when using the *****Ah-cre *****and *****Vil-Cre-ER^T2 ^*****Systems for Conditionally Deleting *****CatnB *****in Similar Numbers of ISCs within the Small Intestine Epithelia. ** (a&b) H&E stained formalin fixed sections indicating location of crypt height ([), an apoptotic (←) and mitotic cell (↓). Quantification of the average number of cells per crypt between wild type (blue) and *CatnB^flox^* (orange) mice at three time points (d.p.i.) (c) crypt height, (d) mitosis and (e) apoptosis (error bars indicate standard deviation). Panel (c)-(e) modified from Parry *et al*^16^. Please click here to view a larger version of this figure.


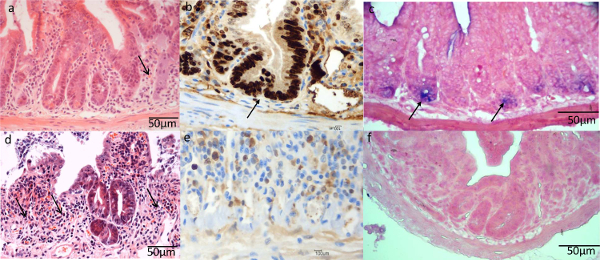
**Figure 4: Comparison of the ISC Characteristics using *****Ah-cre *****and *****Vil-Cre-ER^T2 ^*****Systems for Conditionally Deleting *****CatnB *****in the Small Intestine Epithelia.** Epithelial crypt cells 3 days after deletion of *CatnB *using the* Ah-cre* (a-c) or *vil-Cre-ER^T2 ^*(d-f) system. (a&d) H&E section showing areas of crypt loss; (b&d) Ki-67 IHC demonstrating loss of proliferative cells using *vil-Cre-ER^T2 ^*; (c&f) *Olfm4 in situ *demonstrating presence of functional ISCs using *Ah-cre*. Panel (a)-(f) modified from Parry *et al*^16^. Please click here to view a larger version of this figure.


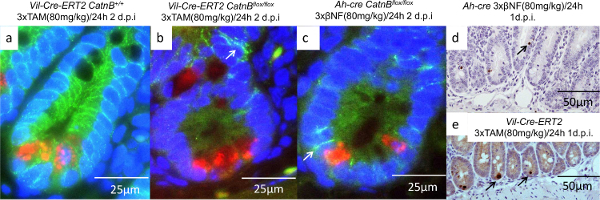
**Figure 5: Characterization of the Paneth Cells after *****CatnB *****Deletion using the *****Vil-Cre-ER^T2 ^*****and *****Ah-cre *****Systems.** (a-c): Immunofluorescence images of crypts showing Paneth cell (red), B-catenin (green) and nucleus (blue), arrow indicate membrane bound β-catenin; (d-e) IHC for Caspase-3 indicating apoptotic Paneth cells are absent in the *Ah-cre *(d)* but present in the vil-Cre-ER^T2 ^*system (e). Panel (a)-(e) modified from Parry *et al*^16^. Please click here to view a larger version of this figure.

**Table:** 

Acetate buffer	*	*	To make 100 ml: 4.8 ml 0.2 M Acetic acid, 45.2 ml 0.2 M Sodium acetate & 50 ml distilled water
Acetic acid	Fisher Scientific	C/0400/PB17	
Acetic anhydride	Sigma	A6404	
Acetic anhydride solution	*	*	2 M Acetic anhydride in 0.1 M triethanolamine hydrochloride
Alcian Blue	Sigma	A5268	
Alcian Blue PH 2.5	*	*	To make 500 ml: 15 ml acetic acid, 5 g Alcian Blue & 485 ml distilled water
anti-digoxigenin alkaline phosphatase conjugated antibody	Abcam	ab119345	
B(beta)-Naphthoflavone	Sigma	N3633	BNF, inject without allowing the solution to cool too much as compound will drop out of solution. Solution can be re-used – store at -20 °C between uses, do not reheat more than twice.
Bloxall	Vector Labs	SP-6000	
BM purple	Roche	11442074001	
BSA	Sigma	A4503	Bovine serum albumin
Chloroform	Fisher Scientific	C/4920/17	
Citrate Buffer/Antigen Unmasking Solution	Vector Labs	H-3300	
Corn oil	Sigma	C8627	
Demucifiying solution	*	*	For 500 ml: 50 ml glycerol, 50 ml Tris 0.1M pH8.8, 100 ml EtOH, 300 ml saline (0.9% NaCl in water), DTT 1.7 g. Demucifying solution can be made in advance and stored, but DTT sholud be added just before incubation (340 mg/100 ml).
DEPC treated water	Life Technologies	750023	
DTT	Sigma	101509944	
EDTA	Sigma	O3690	0.5 M
Ethanol	Fisher Scientific	E/0650DF/17	
Filter paper	Whatman	3000917	
Formaldehyde	Sigma	F8775	
Formalin	Sigma	SLBL11382V	Neutral buffered formalin
Formamide	Sigma	F5786	
Glutaraldehye	Sigma	G6257	
H_2_O_2_	Sigma	216763	
Haematoxylin	Raymond A Lamb	12698616	
HBSS	Gibco	14175-053	HBSS (-MgCl2+; -CaCl2)
Hybridization buffer	*	*	5× SSC, 50% formamide, 5% SDS, 1 mg/ml heparin, 1 mg/ml calf liver tRNA
Hydroquinone	Sigma	H9003	
ImmPACT DAB Peroxidase	Vector Labs	SK-4105	
Immpress HRP Anti-Mouse IgG Kit	Vector Labs	MP-7402	
Immpress HRP Anti-Rabbit IgG Kit	Vector Labs	MP-7401	
Intestinal tissue powder	*	*	The small intestines of 5 adult mice were combined and homogenized in the minimum volume of ice cold PBS. 4 volumes of ice cold acetone were added to the homogenized intestine, which was mixed thoroughly and incubated on ice for 30 min. This was centrifuged and the pellet was washed using ice cold acetone. This was further centrifuged and the resulting pellet spread onto filter paper and allowed to dry. Once thoroughly dry the material was ground to a fine powder using a pestle and mortar.
K-ferricyanide	Sigma	P-3667	
K-ferrocyanide	Sigma	P3289	
Levamisole	Sigma	L0380000	
Methacarn	*	*	60% Methanol:30% Chloroform:10% Acetic acid
Methanol	Fisher Scientific	M/4000/17	
MgCl2	Sigma	M8266	
Normal goat serum	Vector Labs	S-1012	NGS
Normal rabbit serum	Dako	X0902	NRS
NTMT	*	*	100 mM NaCl, 100 mM Tris HCl, 50 mM MgCl2, 0.1% Tween20, 2 mM Levamisole
PAP pen	Vector	H-400	
Paraformaldehyde	Sigma	P6148	
PBT	*	*	0.5 M NaCl, 10 mM TrisHCL pH 7.5, 0.1% Tween 20
Penicillin/Streptomycin	Gibco	15140-122	100x solutiuon.
Phosphate buffered saline (10x)	Fisher Scientific	BP3994	Dilluted 1:10 with distilled water to make 1x
PLL slides	Sigma	P0425-72EA	Poly-L-lysine microscope slides
Proteinase K	Sigma	P2308	
Proteinase k solution	*	*	Dilute Proteinase K at 200 µg/ml in 50 mM Tris, 5 mM EDTA.
Ralwax	BDH	36154 7N	
Reducer Solution	*	*	To make 100 ml: 1 g Hydroquinone, 5 g sodium sulphite & 100 ml distilled water
RnaseA	Sigma	R6148	
Saline	*	*	0.9% NaCl in distilled water
SDS	Sigma	I3771	
Sheep serum	Sigma	S3772	
Silver nitrate	Sigma	S/1240/46	
Silver solution	*	*	To make 100 ml: 10 ml Acetate buffer, 87 ml distilled water, 3 ml 1% silver nitrate
Sodium acetate	Fisher Scientific	S/2120/53	
Sodium Chloride	Sigma	S6753	NaCl
Sodium sulfite	Sigma	239321	
SSC	Sigma	93017	20x saline sodium citrate
Surgical tape	Fisher Scientific	12960495	
Tamoxifen	Sigma	T5648	TAM, inject without allowing the solution to cool too much as compound will drop out of solution. Solution can be re-used – store at -20 °C between uses, do not reheat more than twice.
TBS/T	Cell Signalling	#9997	
Triethanolamine hydrochloride	Sigma	T1502	
Tris-HCL	Invitrogen	15567-027	
Tween20	Sigma	TP9416	
VectaMount	Vector Labs	H-5000	
VectaShield Hardset mounting Medium with DAPI	Vector Labs	H-1500	
Vectastain ABC Kit	Vector Labs	PK-4001	
X-gal	Promega	V3941	
X-gal fixative	*	*	2% formaldehyde, 0.1% glutaraldehyde in 1xPBS
X-gal stain	*	*	X-gal stain; 200 μl X-gal (A) in 50 ml solution B (0.214 g MgCl2, 0.48 g K-ferricyanide, 0.734 g K-ferrocyanide in 500 ml PBS). Solution B can be made up oin advance and stored at 4 °C
Xylene	Fisher Scientific	X/0200/21	

**Table 1:** **Materials and Methods**

**Table:** 

Target	beta-Catenin	Lysozyme	Ki67	Caspase-3	Villin
Commercial source of primary Ab	Transduction Labs	Neomarkers	Vector Labs	R&D Systems	Santa Cruz
Catalogue Number	610154	RB-372	VP-K452	AF835	SC-7672
Primary Ab raised in	Mouse (mAb)	Rabbit (pAb)	Mouse (mAb)	Rabbit (pAb)	Goat (pAb)
Antigen retrieval	Boiling water bath/Citrate Buffer	Boiling water bath/Citrate buffer	Boiling water bath/Citrate buffer	Boiling water bath/Citrate buffer	Boiling water bath/Citrate buffer
Peroxidase block	Bloxall or 2% H_2_O_2_, 45 sec	Bloxall or 1.5% H_2_O_2_, 30 min	Bloxall or 0.5% H_2_O_2_, 20 min	Bloxall or 2% H_2_O_2_, 45 sec	Bloxall or 3% H_2_O_2_, 20 min
Serum block	1% BSA, 30 min	10% NGS, 30min	20% NRS, 20 min	10% NGS, 45 min	10% NRS, 30 min
Wash buffer	PBS	TBS/T	TBS/T	PBS	TBS/T
Conditions for primary Ab	1/300, 2 hr at RT	1/100, 1hr at RT	1/50, 1hr at RT	1/750, o/n at 4°C	1/500, 1hr at RT
Secondary Ab	Immpress HRP Anti-Mouse IgG Kit	Immpress HRP Anti-Rabbit IgG Kit	Biotinylated Rabbit anti-Mouse	Biotinylated Goat anti-Rabbit	Biotinylated Rabbit anti-Goat
Conditions for secondary Ab	1 hr at RT	30min at RT	1/200, 30 min at RT	1/200, 30 min at RT	1/200, 30 min at RT
Signal amplification	N/A	N/A	ABC kit	ABC kit	ABC kit
Signal detection	ImmPACT DAB Peroxidase	ImmPACT DAB Peroxidase	ImmPACT DAB Peroxidase	ImmPACT DAB Peroxidase	ImmPACT DAB Peroxidase
Immunofluorescence antibody	Alexafluor 488	Alexafluor 594	N/A	N/A	N/A
Immunofluorescence Properties	Excitation Max 488/Emission Max 525	Excitation Max 595/Emission Max 617			
Common Filter Set	FITC	Texas Red			

**Table 2: IHC Antibodies and Conditions**

## Discussion

Using conditional *cre-lox* transgenic mice to dissect the function of genes and cells is a commonly used approach. These models have been used with great success in the intestine to identify and characterize the stem cells^2,4-6^ and understand their role in disease^25^. To fully exploit these models requires a comprehensive characterization of the system to enable data to be interpreted correctly. A complete understanding of these systems is difficult to achieve due to genes rarely being specific to a solitary cell type or location, a lack of biological knowledge and inefficiency of the systems used to induce *Cre* expression. The methods described here demonstrate how we overcome these issues through experimental design and application of existing knowledge. Although we used these methods to answer a specific research question the techniques presented here are generic and can be exploited for any research investigating the murine intestine.

**Preparation of Intestinal Tissue**

The crucial step for ensuring robust results is the harvesting and processing of the tissue, which needs to be processed in a timely manner and fixation protocols strictly adhered to. As almost all significant issues downstream can be attributed to artefacts associated with the tissue drying out and/or incomplete fixation. Timing is crucial to prevent degradation of tissue architecture and/or nucleic acids and proteins. Incomplete or overzealous fixation can result in loss of histochemical resolution. Incomplete fixation due to insufficient time or sections too thick to allow fixative penetration can result in loss of resolution within the intestinal crypts that can be observed as a “tide mark” upon IHC analysis. Further it is crucial that fixation does not extend for too long, as nuclear β-catenin can diffuse out of the nucleus unless immediately processed and wax embedded following formalin fixation.

**Role of Paneth Cells in the ISC Niche**

The data presented here effectively show the importance of Paneth cells in crypt regeneration in the adult intestine following ISC loss. However there remained the possibility that *Ah-cre *spares a population of ISCs that *vil-Cre-ER^T2 ^*targets. Tian *et al.^26^* elegantly demonstrated that the *Lgr5^hi ^*ISCs are replaced by a population of *Lgr5^lo ^*reserve ISCs. It now seems likely that these ISCs are spared in the *Ah-cre *system due to the reserve population having been identified as secretory cell precursors^6,7^. The importance of the mature Paneth cell in supporting these secretory cell precursors when required to revert to an ISC state remains to be answered. As Paneth cells constitute the ISC niche^8^ and play roles in regulating the ISC responses to calorie intake^27^ and inflammation^28^ it remains likely that their nursing functions will extend to their own precursors.

**New Approaches and Technologies to Effectively Model Human Colorectal Cancer**

The discovery of the ISC led to the identification of genes which are now being used to generate new mouse models for investigating the roles of genes and cells in intestinal biology and disease, reviewed by Clarke *et al^9^*. The only limitations to this technique is the identification of genes to express the Cre protein. Currently ISCs are routinely investigated using conditional transgenic mice based on the *Lgr5 *gene expression pattern. Mice which express *Cre *from the *Lgr5 *promoter have been used to delete *Apc*, the gene most commonly mutated in colorectal cancer (CRC), demonstrating the ISC as the cell of origin^25^. Selectively deleting other CRC genes in these cells is providing insight into disease progression and spread *e.g*. *PTEN*^29^. Further insight into ISC function is being retrieved by specifically ablating *Lgr5*-expressing cells in mice using a human diphtheria toxin receptor (DTR) gene knocked into the *Lgr5* locus^26^. Other strategies use the Tet-O system which enables ongoing reversible expression of mutant proteins^30^. Using these tools to modify gene(s) in different cells^31^ and locations^32,33^ is used to understand how cancer initiate, progress and metastasize^34^. Alternatively mutagenesis using the sleeping beauty transposon system is identifying new drivers of CRC. The continuing development of mice, techniques and genetic alteration strategies is continuing to develop more patient relevant models.

New methods have been developed for characterization of the intestinal epithelia and ISCs. Characterization of the ratio of epithelial cell types can be achieved using flow cytometry based on the differential expression of lectin and CD24^35^. Potentially the biggest progress in understanding ISC biology and their role in disease will be made using the *ex vivo *organoid culture system^36^. This system allows normal and malignant ISCs to culture in 3D, where they replicate and differentiate in a more physiologically relevant way. It is hoped that these will enable direct testing of drugs on patient samples *in vitro, *paving the way for personalized medicine^37^.

## Disclosures

The authors have nothing to disclose.

## References

[B0] Cheng H, Origin Leblond CP (1974). differentiation and renewal of the four main epithelial cell types in the mouse small intestine. V. Unitarian Theory of the origin of the four epithelial cell types. Am J Anat.

[B1] Barker N (2007). Identification of stem cells in small intestine and colon by marker gene Lgr5. Nature.

[B2] Clevers H (2015). The gut, a clonal conveyor belt.

[B3] Snippert H (2010). Intestinal crypt homeostasis results from neutral competition between symmetrically dividing Lgr5 stem cells. Cell.

[B4] Lopez-Garcia C, Klein AM, Simons BD, Winton DJ (2010). Intestinal stem cell replacement follows a pattern of neutral drift. Science.

[B5] Buczacki SJ (2013). Intestinal label-retaining cells are secretory precursors expressing Lgr5. Nature.

[B6] Basak O (2014). Mapping early fate determination in Lgr5+ crypt stem cells using a novel Ki67-RFP allele. EMBO J.

[B7] Sato T (2011). Paneth cells constitute the niche for Lgr5 stem cells in intestinal crypts. Nature.

[B8] Young M, Ordonez L, Clarke AR (2013). What are the best routes to effectively model human colorectal cancer. Molecular oncology.

[B9] Sauer B, Henderson N (1988). Site-specific DNA recombination in mammalian cells by the Cre recombinase of bacteriophage P1. Proc Natl Acad Sci U.S.A.

[B10] Brault V (2001). Inactivation of the beta-catenin gene by Wnt1-Cre-mediated deletion results in dramatic brain malformation and failure of craniofacial development. Development.

[B11] Fevr T, Robine S, Louvard D, Huelsken J (2007). Wnt/β-catenin is essential for intestinal homeostasis and maintenance of intestinal stem cells. Molecular and Cellular Biology.

[B12] Ireland H (2004). Inducible Cre-mediated control of gene expression in the murine gastrointestinal tract: effect of loss of beta-catenin. Gastroenterology.

[B13] Kemp R (2004). Elimination of background recombination: somatic induction of Cre by combined transcriptional regulation and hormone binding affinity. Nucleic Acids Res.

[B14] Madison BB (2002). Cis elements of the villin gene control expression in restricted domains of the vertical (crypt) and horizontal (duodenum, cecum) axes of the intestine. J Biol Chem.

[B15] Parry L, Young M, El Marjou F, Clarke AR (2013). Evidence for a crucial role of paneth cells in mediating the intestinal response to injury. Stem Cells.

[B16] Soriano P (1999). Generalized lacZ expression with the ROSA26 Cre reporter strain. Nat Genet.

[B17] el Marjou F (2004). Tissue-specific and inducible Cre-mediated recombination in the gut epithelium. Genesis.

[B18] Guillemot F, Nagy A, Auerbach A, Rossant J, Joyner AL (1994). Essential role of Mash-2 in extraembryonic development. Nature.

[B19] Gregorieff A (2005). Expression pattern of Wnt signaling components in the adult intestine. Gastroenterology.

[B20] van der Flier LG, Haegebarth A, Stange DE, van de Wetering M, Clevers H (2009). OLFM4 is a robust marker for stem cells in human intestine and marks a subset of colorectal cancer cells. Gastroenterology.

[B21] Merritt A, Allen T, Potten C, Hickman J (1997). Apoptosis in small intestinal epithelial from p53-null mice: evidence for a delayed, p53-independent G2/M-associated cell death after gamma-irradiation. Oncogene.

[B22] Marshman E, Ottewell P, Potten C, Watson A (2001). Caspase activation during spontaneous and radiation-induced apoptosis in the murine intestine. J Pathol.

[B23] Fevr T, Robine S, Louvard D, Huelsken J (2007). Wnt/beta-catenin is essential for intestinal homeostasis and maintenance of intestinal stem cells. Mol Cell Biol.

[B24] Barker N (2009). Crypt stem cells as the cells-of-origin of intestinal cancer. Nature.

[B25] Tian H (2011). A reserve stem cell population in small intestine renders Lgr5-positive cells dispensable. Nature.

[B26] Yilmaz OH (2012). mTORC1 in the Paneth cell niche couples intestinal stem-cell function to calorie intake. Nature.

[B27] Adolph TE (2013). Paneth cells as a site of origin for intestinal inflammation. Nature.

[B28] Marsh V (2008). Epithelial Pten is dispensable for intestinal homeostasis but suppresses adenoma development and progression after Apc mutation. Nat Genet.

[B29] Jardé T (2013). In vivo and in vitro models for the therapeutic targeting of Wnt signaling using a Tet-OΔN89β-catenin system. Oncogene.

[B30] Hung KE (2010). Development of a mouse model for sporadic and metastatic colon tumors and its use in assessing drug treatment. Proc Natl Acad Sci U S A.

[B31] Dacquin R, Starbuck M, Schinke T, Karsenty G (2002). Mouse alpha1(I)-collagen promoter is the best known promoter to drive efficient Cre recombinase expression in osteoblast. Dev Dyn.

[B32] Hinoi T (2007). Mouse model of colonic adenoma-carcinoma progression based on somatic Apc inactivation. Cancer Res.

[B33] Sansom OJ (2006). Loss of Apc allows phenotypic manifestation of the transforming properties of an endogenous K-ras oncogene in vivo. Proc Natl Acad Sci U.S.A.

[B34] Wong VW (2012). Lrig1 controls intestinal stem-cell homeostasis by negative regulation of ErbB signalling. Nat Cell Biol.

[B35] Sato T (2009). Single Lgr5 stem cells build crypt-villus structures in vitro without a mesenchymal niche. Nature.

[B36] van de Wetering M (2015). Prospective Derivation of a Living Organoid Biobank of Colorectal Cancer Patients. Cell.

